# Targeted exon skipping rescues ciliary protein composition defects in Joubert syndrome patient fibroblasts

**DOI:** 10.1038/s41598-019-47243-z

**Published:** 2019-07-25

**Authors:** Elisa Molinari, Simon A. Ramsbottom, Shalabh Srivastava, Philip Booth, Sumaya Alkanderi, Seamus M. McLafferty, Laura A. Devlin, Kathryn White, Meral Gunay-Aygun, Colin G. Miles, John A. Sayer

**Affiliations:** 1 Institute of Genetic Medicine, Newcastle University, International Centre for Life, Central Parkway, Newcastle upon Tyne, NE1 3BZ United Kingdom; 2 Renal Services, The Newcastle Hospitals NHS Foundation Trust, Newcastle upon Tyne, NE7 7DN United Kingdom; 3 EM Research Services, Newcastle University, Newcastle upon Tyne, United Kingdom; 4 Medical Genetics Branch, National Human Genome Research Institute, National Institutes of Health, Bethesda, MD 20892 USA; 5 Department of Pediatrics, McKusick-Nathans Institute of Genetic Medicine, Johns Hopkins University School of Medicine, Baltimore, MD 21205 USA; 6 NIHR Newcastle Biomedical Research Centre, Newcastle upon Tyne, United Kingdom

**Keywords:** Paediatric kidney disease, Antisense oligonucleotide therapy, Mechanisms of disease

## Abstract

Joubert syndrome (JBTS) is an incurable multisystem ciliopathy syndrome. The most commonly mutated gene in JBTS patients with a cerebello-retinal-renal phenotype is *CEP290* (alias *JBTS5*). The encoded CEP290 protein localises to the proximal end of the primary cilium, in the transition zone, where it controls ciliary protein composition and signalling. We examined primary cilium structure and composition in fibroblast cells derived from homozygous and compound heterozygous JBTS5 patients with nonsense mutations in *CEP290* and show that elongation of cilia, impaired ciliogenesis and ciliary composition defects are typical features in JBTS5 cells. Targeted skipping of the mutated exon c.5668 G > T using antisense oligonucleotide (ASO) therapy leads to restoration of CEP290 protein expression and functions at the transition zone in homozygous and compound heterozygous JBTS5 cells, allowing a rescue of both cilia morphology and ciliary composition. This study, by demonstrating that targeted exon skipping is able to rescue ciliary protein composition defects, provides functional evidence for the efficacy of this approach in the treatment of JBTS.

## Introduction

Joubert Syndrome (JBTS) is an incurable multisystem condition, in most cases inherited as an autosomal recessive disease. The diagnostic hallmark of JBTS patients is a characteristic cerebellar malformation, known as the ‘molar tooth sign’, which is accompanied by hypotonia, ataxia, oculo-motor apraxia, global developmental delay and breathing dysregulation. Such neurological defects can variably be associated with multi-organ involvement leading to retinal degeneration, renal and liver disease.

To date, more than 30 genes have been associated with JBTS, all of which encode for proteins that are implicated in ciliary structure, assembly and/or function. Primary cilia are non-motile microtubule-based specialized organelles that protrude from the surface of many quiescent or post-mitotic eukaryotic cells. The primary cilium acts as an important signalling centre of the cell; its membrane is enriched in receptors and signalling molecules and its tightly-regulated protein composition makes it a specialized compartment within the cell, where extensive cross-talk between different signalling pathways takes place^1,2^. The protein composition within the cilium is controlled by the transition zone (TZ), a distinct domain that acts as a diffusion barrier at the distal end of the basal body^3–5^ and is defined by the presence of Y-shaped electron dense structures that connect the axonemal microtubule doublets and the ciliary membrane^6^. Two distinct protein complexes form the TZ, the NPHP module and the MKS module that are composed of proteins mutated mainly in nephronophthisis and Meckel-Gruber syndrome, respectively. Loss of function studies on the components of these complexes revealed that the TZ is essential for ciliogenesis and for the control of ciliary composition^7^. Absence of JBTS proteins from the TZ can affect ciliary membrane composition with dramatic effects on ciliary enrichment of important signalling proteins such as smoothened (SMO) and adenylate cyclase 3 (AC3)^4^. It has been proposed that perturbations of TZ architecture cause JBTS^5,8^.

An important hub within the TZ protein network is CEP290, a 290 kDa centrosomal protein, which is part of the MKS complex but also interacts with NPHP complex components^9,10^. Electron microscopy studies based on immuno-gold labelling of cep290 in *Chlamydomonas reinhardtii* showed that cep290 associates with the TZ links and localises between the outer doublet microtubules and the membrane of the transition zone^11^ where it is thought to link these two components by binding the ciliary membrane through its N-terminus and the microtubules through a C-terminal domain^12^. CEP290 is also found associated with centriolar satellites where it interacts with their main component, PCM-1. Both CEP290 and PCM-1 are implicated in ciliogenesis through the recruitment to the primary cilium of the small GTPase, BBS complex interactor, RAB8^13,14^.

*CEP290* (*JBTS5*) is the most commonly mutated gene in JBTS patients with a cerebello-retinal-renal phenotype^15,16^. In the absence of a full-length CEP290 protein, JBTS cells display reduced ciliogenesis and fibroblasts from one patient were shown to have reduced ciliary levels of SMO and AC3^17^.

We have recently demonstrated that antisense oligonucleotide (ASO)-mediated skipping of the mutated exon is able to restore *CEP290* expression and rescues cilia length defects in urine derived renal epithelial cells (URECs) from a JBTS5 patient with homozygous mutations in *CEP290* (c.5668 G > T; p.(G1890X))^18^.

Here we analyse the ciliary phenotype of fibroblast cells from a homozygote and a heterozygote carrier of a premature stop codon p.(G1890X) in CEP290 and show that cilia elongation and aberrant ciliary protein composition are typical features in JBTS5 cells. We demonstrate that ASO-mediated skipping of mutated exon 41 rescues not only elongated ciliary phenotypes, as previously shown in URECs^18^, but also ciliary composition defects, providing further functional evidence for the use of this therapeutic approach to treat JBTS. Importantly, skipping of the premature stop codon on one allele in fibroblasts from a compound heterozygote patient is sufficient to rescue ciliary defects.

## Results

### Reduced CEP290 protein levels in JBTS5 fibroblasts lead to ciliary defects

We generated fibroblast lines from skin biopsy specimens obtained from a JBTS5 patient (P1, referred to as JBTS-AA in our URECs studies)^18^ with a homozygous nonsense mutation c.5668 G > T; p.(G1890X) in *CEP290* and an unrelated wild type control (C1). We also derived fibroblasts from a JBTS5 patient (P2) with compound heterozygous mutations in *CEP290* and from a control heterozygote (C2; patient P2 is referred to as JSRD-2, control C2 as Ctrl-4, respectively, in a related fibroblast study)^17^. P2 and C2 are both heterozygote for the c.5668 G > T allele; the other *CEP290* allele in P2 harbours a truncating frameshift mutation (Supplementary Table S1). Indeed, c.5668 G > T; p.(G1890X) is a frequent mutation within *CEP290*, representing the 16% of all *CEP290* mutations (https://cep290base.cmgg.be).

**Figure 1:**
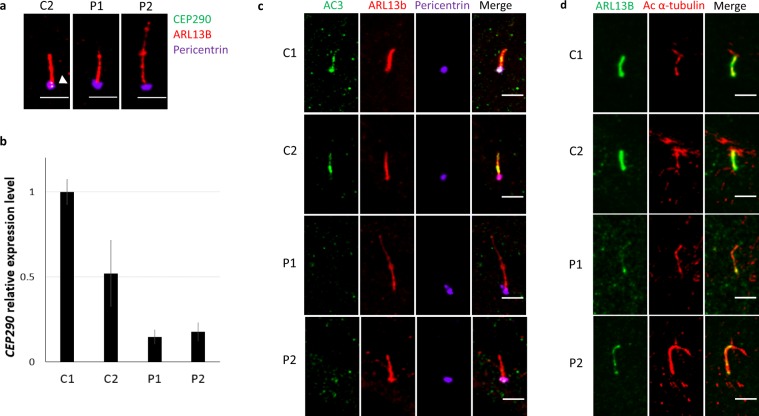
Reduced CEP290 protein expression leads to defects in localisation of ciliary membrane proteins AC3 and ARL13B. (**a**) Representatisve immunofluorescence images of control heterozygote fibroblasts C2 and JBTS5 fibroblasts P1 and P2, serum starved for 48 h. CEP290 protein is visible at the base of cilium in control fibroblasts, but is not detectable in P1 and P2. Green - CEP290, Red - ARL13B, Violet - pericentrin. Scale bar 5 μm. (**b**) *CEP290* gene expression analysis of fibroblasts obtained from wild type control fibroblasts C1, control heterozygote fibroblasts C2 and JBTS5 fibroblasts P1 and P2, serum starved for 48 h. Expression level of CEP290 is reduced by more than 80% in patients fibroblasts compared to the wild type control, indicating that mutant transcripts are efficiently degraded. Each bar represents the mean value from three replicates. Values are normalised to wild type control C1. (**c**) Representative immunofluorescent micrographs of control wild type fibroblasts C1, control heterozygote fibroblasts C2 and JBTS5 fibroblasts P1 and P2, serum starved for 48 h. Ciliary AC3 levels are visibly reduced in JBTS5 fibroblasts. Green – AC3, Red – ARL13B, Violet – pericentrin. Scale bar 5 μm. (**d**) Representative immunofluorescence micrographs of control wild type fibroblasts C1, control heterozygote fibroblasts C2 and JBTS5 fibroblasts P1 and P2, serum starved for 48 h. Ciliary ARL13B levels are visibly reduced in JBTS5 fibroblasts. Green – ARL13B, Red – acetylated α-tubulin. Scale bar 5 μm.

CEP290 wild type protein has been reported to localise at the TZ as well as at centriolar satellites^11,14^. Immunofluorescence analysis revealed that, whilst in control fibroblasts CEP290 localises mainly to the proximal end of the primary cilium, in patient fibroblasts, P1 and P2, no full-length CEP290 protein was detectable at the transition zone, or elsewhere in the cell (Fig. 1a, Supplementary Fig. S1).

A premature stop codon can result either in the production of a truncated protein or in gene knockdown, as the aberrant transcript may be degraded by nonsense-mediated decay. Consistently with that previously described in URECs^18^, qPCR assay shows that *CEP290* transcript abundance in P1 fibroblasts was dramatically reduced to 15% of the expression observed in wild type C1 cells. We detected a similar reduction to 18% of *CEP290* transcript in patient P2 fibroblasts compared to wild type cells, indicating that mRNA transcripts from mutated alleles are unstable in these cells. *CEP290* expression levels in heterozygote control C2 c.5668 G > T; p.(G1890X) were reduced to approximately 50% compared to wild type cells C1 (Fig. 1b).

Absence of TZ proteins leads to ciliary composition defects^4^. TZ abnormalities, that result in aberrant cilia composition, have been proposed to be at the basis of JBTS^5,8^. We wondered whether defects in ciliary composition are a typical feature in JBTS5 patient cells.

Defects in the transport of the ciliary protein AC3 were previously described in a single JBTS5 patient^17^. Immunofluorescence analysis revealed that loss of CEP290 protein expression results in a reduced ciliary localisation, but not reduced total protein levels, of the ciliary protein AC3 (Fig. 1c, Supplementary Fig. S1) in both P1 and P2 cell lines. Moreover, Shimada *et al*. recently described low levels of JBTS protein ADP Ribosylation Factor-Like GTPase 13B (ARL13B) in the cilium in P2 JBTS5 fibroblasts, as opposed to total protein levels, suggesting incorrect trafficking of ARL13B to the cilium^17^. We confirmed reduced ciliary localisation of ARL13B in P2 fibroblasts and observed a similar reduction in P1, not accompanied by a reduction in total protein expression or stability, as revealed by western blot, indicating that defects in the localisation of ciliary membrane proteins to the cilium are a common feature in JBTS5 cells (Fig. 1d, Supplementary Fig. S1).

**Figure 2:**
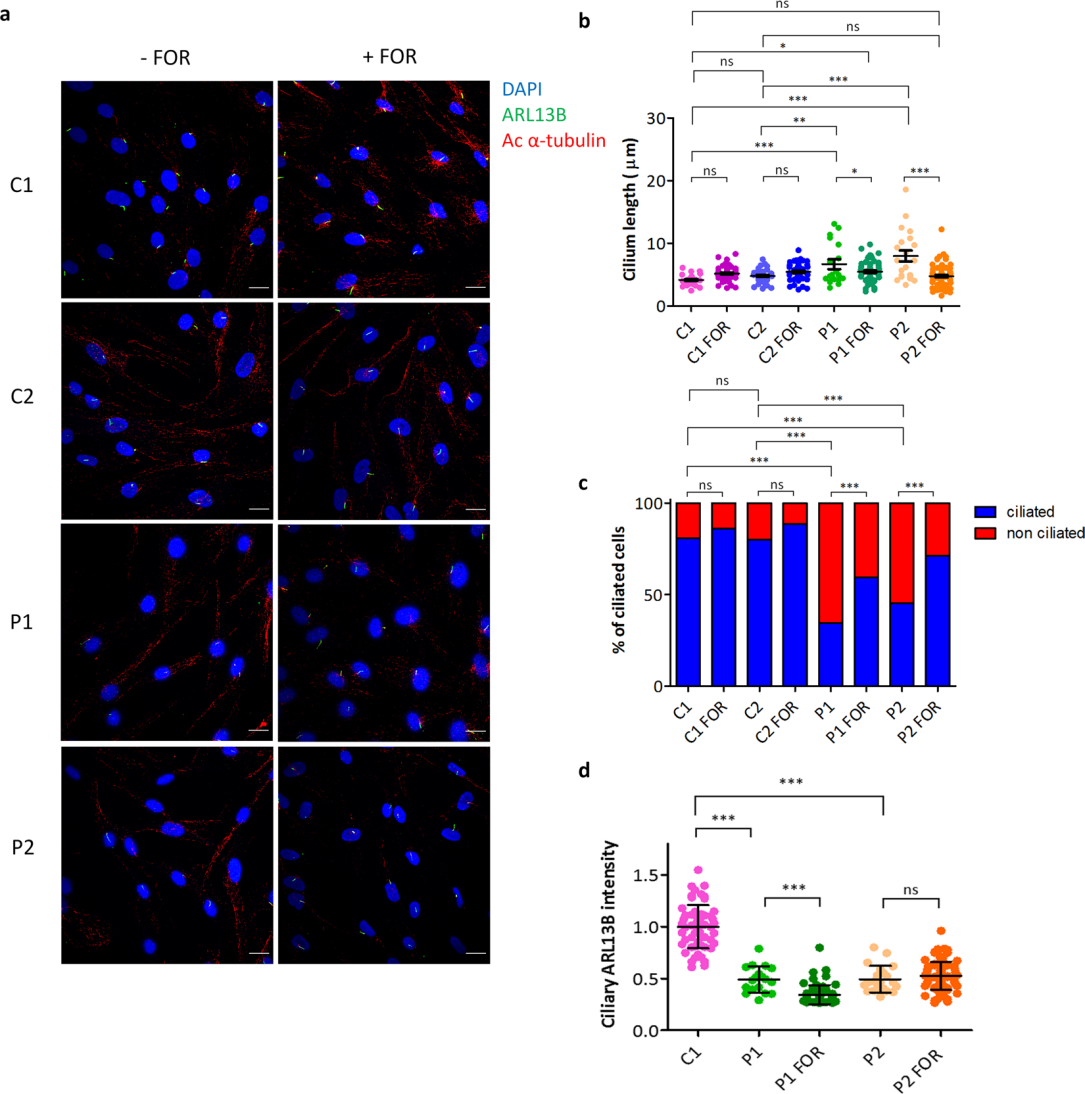
Treatment with forskolin results in a rescue of cilia biogenesis and elongation defects but not of ciliary localisation of ARL13B in JBTS5 cells. (**a**) Immunofluorescence microscopy of control fibroblast lines C1, C2 and JBTS5 lines P1, P2. Untreated, serum starved cells (left panel) and cells treated with 25 μM forskolin (right panel). Treatment with forskolin results in an increase of ciliated cells upon serum starvation and in a rescue of ciliary phenotype in JBTS5 cells. DAPI- blue (Nuclei), Green - ARL13B, Red - Acetylated α-tubulin. Scale bar 20 μm. (**b**) Quantification of cilia length in control fibroblast lines C1, C2 and JBTS5 lines P1, P2, untreated or treated with forskolin. Mean length of JBTS5 fibroblast cilia decreased from 6.7 μm to 5.5 μm and 8.0 μm to 4.8 μm in P1 and P2, respectively, after treatment with forskolin. *P < 0.05, **P < 0.01, ***P < 0.001, ns, non-significant, one-way ANOVA, n = 341. (**c**) Quantification of percentage of ciliated cells in control fibroblast lines C1, C2 and JBTS5 lines P1, P2, untreated or treated with forskolin. Percentage of ciliated JBTS5 fibroblasts increased from 34% to 59% and from 45% to 71% in P1 and P2, respectively, after treatment with forskolin. ***P < 0.001, ns, non-significant, Fisher’s exact test. Bonferroni correction was used to adjust for multiple comparisons, n = 1244. (**d**) Quantification of ciliary ARL13B intensity normalised to the background in control fibroblast lines C1 and JBTS5 lines P1 and P2, untreated or treated with forskolin. Values are expressed as ARL13B ciliary intensity relative to ciliary intensity in C1 cells. ***P < 0.001, ns, non-significant, one-way ANOVA, n = 239. FOR, forskolin.

Electron microscopy and immunofluorescence analysis (where cilia were identified using both ARL13B and acetylated α-tubulin staining) revealed that CEP290 loss at the TZ also results in a reduced percentage of cells that display a primary cilium after serum starvation and in increased average cilia length in JBTS5 fibroblasts (Supplementary Fig. S2), as previously described^17^. It has been reported that AC3 inhibition results in increased ciliary elongation^19^, which implies that AC3 contributes directly or indirectly to the regulation of primary cilia length. Interestingly, treatment of patient fibroblasts P1 and P2 with adenylate cyclase (AC) allosteric agonist forskolin resulted in a rescue of cilia elongation (Fig. 2a,b) and biogenesis (Fig. 2a,c) but not of ciliary localisation of ARL13B (Fig. 2d).

### ASO-induced skipping of mutated exon 41 rescues CEP290 protein expression

**Figure 3:**
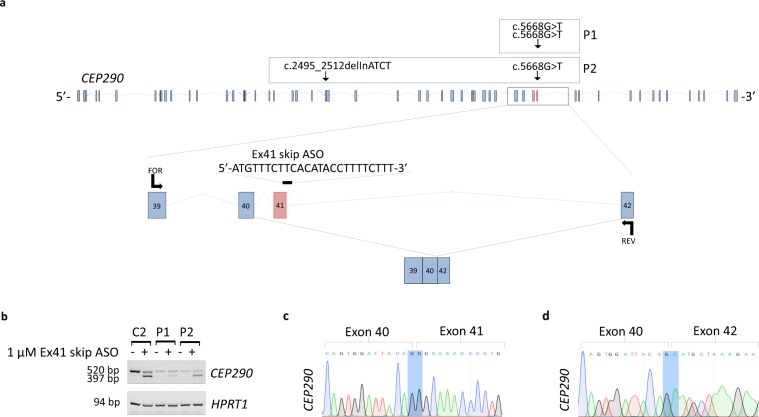
Ex41 skip ASO treatment is able to induce targeted skipping of exon 41 from *CEP290* transcript. (**a**) Schematic of human CEP290, consisting of 54 exons. In red, exon 41. The full-length mRNA transcript consists of 7951 nucleotides, translated into a protein of 2479 amino acids. JBTS5 patient fibroblasts harbour a nonsense mutation in the *CEP290* gene (c.5668 G > T; p.(G1890X)) leading to a premature stop codon and truncation of the reading frame within exon 41 as shown. Mutation on the other *CEP290* allele in P2 is also shown. A morpholino-based antisense oligonucleotide (Ex41 skip ASO) was designed to block the splice donor site of exon 41 to promote the skipping of exon 41. Skipping the 123 nucleotides of exon 41 leads to a predicted shorter mRNA transcript that encodes a protein consisting of 2438 amino acids. Arrows indicate forward (FOR) and reverse (REV) primers, targeting a site in exon 39 and a site in exon 42, respectively, used for RT-PCR. (**b**) RT-PCR on cDNA isolated from C2, P1 and P2 lysates demonstrates partial efficiency of 1 μM Ex41 skip ASO in skipping 123 bp of exon 41, leading to a shorter *CEP290* mRNA transcript (corresponding to the 397 bp amplicon). Cells treated with 1 μM Ex41 skip ASO produce also a non-skipped *CEP290* transcript (corresponding to the 520 bp product). In untreated cells only a non-skipped amplicon of 520 bp is detectable. *HPRT1* was used as housekeeping gene. (**c**) Sanger sequencing following T-cloning of the 520 bp PCR product from P2 fibroblasts treated with 1 μM Ex41 skip ASO shows presence of exon 41. (**d**) Sanger sequencing following T-cloning of the 397 bp PCR product from P2 fibroblasts treated with 1 μM Ex41 skip ASO shows skipping of exon 41.

JBTS5 patient P1 is homozygous and P2 is compound heterozygous for the *CEP290* mutation c.5668 G > T; p.(G1890X), located within exon 41, a 123 bp exon which begins and ends in the same reading frame. Skipping of this exon would lead to a shorter mRNA transcript that could escape nonsense-mediated degradation leading to the production of a near full-length CEP290 protein. We designed a morpholino antisense oligonucleotide (ASO) that targets the exon 41-intron 41 boundary of human *CEP290* to induce skipping of exon 41 from the transcript (Ex41 skip ASO, Fig. 3a), as previously described^18^. Treatment of control and patient fibroblast cells with 1 µM Ex41 skip ASO for 48 h efficiently induced skipping of exon 41 in a portion of the *CEP290* transcripts, as confirmed by RT-PCR analysis (Fig. 3b) and Sanger sequencing following cloning in pGEM-T Easy Vector of PCR products (Fig. 3c,d). RT-PCR on control and patient fibroblasts treated with 5 μM Ex41 skip ASO shows undetectable levels of *CEP290* transcript retaining exon 41, indicating complete skipping of exon 41 at this dose (Supplementary Fig. S3).

**Figure 4:**
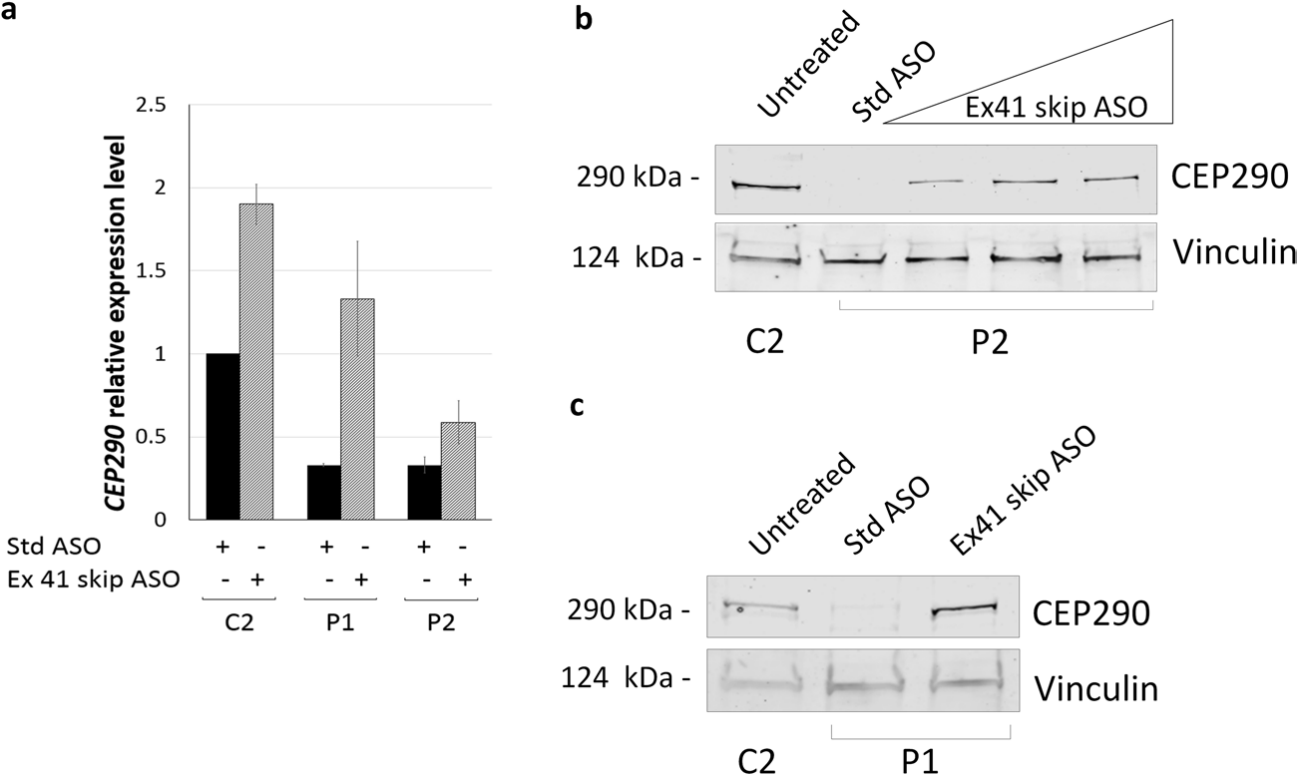
Treatment with Ex41 skip ASO induces the expression of a near full-length CEP290 protein. (**a**) *CEP290* gene expression analysis of fibroblasts obtained from control heterozygote fibroblasts C2 and JBTS5 fibroblasts P1 and P2 treated with 5 μM Std ASO or 5 μM Ex41 skip ASO and serum starved for 48 h. In heterozygote control C2, ASO-induced skipping of exon 41 results in a 2-fold increase of *CEP290* transcript levels. Similarly, treatment with Ex41 skip ASO leads to a 2-fold increase of *CEP290* transcript levels in JBTS5 fibroblasts P2 (59% of *CEP290* expression levels in heterozygote C2 cells) and to a 5-fold increase in JBTS5 fibroblasts P1 (133% of *CEP290* expression levels in heterozygote C2 cells). Each bar represents mean value from three replicates. Values are normalised to heterozygote control C2. (**b**) Western blot demonstrates the presence of full-length CEP290 protein (290 kDa) in C2 protein lysates, whereas protein lysates from P2 cells treated with Std ASO show absence of full-length protein. Treatment with increasing concentrations (1 μM, 5 μM and 10 μM) of Ex41 skip ASO for 48 h results in a rescue of CEP290 protein expression in P2 cells. Vinculin (124 kDa) serves as loading control. (**c**) P1 protein lysates treated with Std ASO show a near absence of full-length protein. *CEP290* exon 41 skipping induced by 5 μM Ex41 skip ASO in P1 cells demonstrates the rescue of CEP290 protein translation to levels higher than in heterozygote C2 cells. Vinculin serves as loading control.

To determine whether Ex41 skip ASO-induced skipping of the nonsense mutation c.5668 G > T within the *CEP290* transcript prevents nonsense-mediated degradation, we measured the levels of *CEP290* mRNA by qPCR. Indeed, *CEP290* transcript levels in patient fibroblasts increased when cells were treated with Ex 41 skip ASO. In patient lines, we observed a 2-fold increase in *CEP290* mRNA in compound heterozygous P2 and a 5-fold increase in *CEP290* mRNA in homozygous P1. Interestingly, in the heterozygote control C2, carrying only a monoallelic change (c.5668 G > T), ASO-induced skipping of exon 41 resulted in a 2-fold increase of *CEP290* transcript levels, compared to cells treated with a standard control morpholino antisense oligonucleotide (Std ASO), indicating that ASO treatment rescued the expression of the heterozygous *CEP290* allele (Fig. 4a).

To assess whether this increase in *CEP290* transcript level was accompanied by an increase in protein and whether this effect was dependent on ASO concentration, we treated P2 cells with increasing concentrations of Ex41 skip ASO and checked CEP290 protein levels by immunoblot. It is worth noting that the antibody used binds to an epitope at the C-terminal of CEP290 protein, meaning that it can only recognise full-length or nearly full-length protein. Full-length CEP290 protein in patient line P2, which was undetectable, dramatically increased when cells were treated with Ex41 skip ASO. We observed an ASO dose-dependent effect when cells were treated with 1 μM and 5 μM Ex41 skip ASO, with no further increase in near full-length protein levels at 10 μM (Fig. 4b). Similarly, in patient line P1, it is possible to distinguish by immunoblot only a very faint band at 290 kDa, which indicates a partial read-through of the nonsense mutation p.(G1890X). However, when patient fibroblasts were treated with 5 μM Ex41 skip ASO, CEP290 protein levels dramatically increased (Fig. 4c).

### Near full-length CEP290 protein lacking exon 41 correctly localises at the TZ

**Figure 5:**
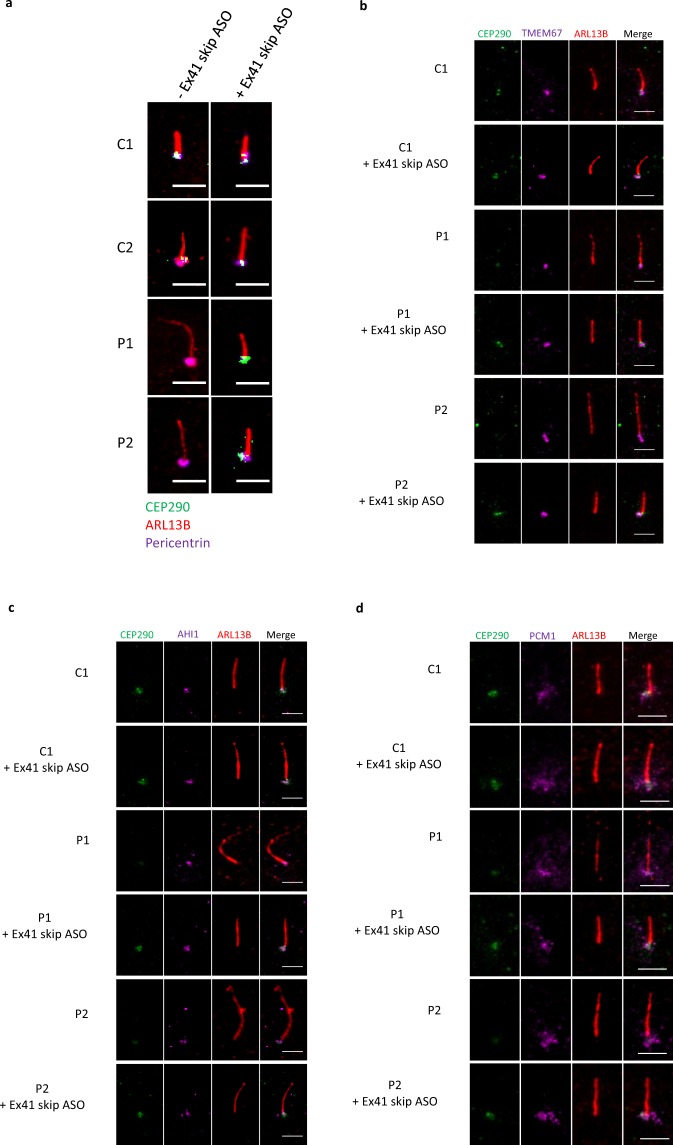
Treatment with Ex41 skip ASO induces the expression of a near full-length CEP290 protein that localises at the TZ. (**a**) Immunofluorescence shows that CEP290 protein is not detectable in untreated P1 and P2 cells. Treatment with 5 μM Ex41 skip ASO in serum-free medium for 48 h rescues CEP290 protein expression which localises to the base of the cilium, in P1 and P2 cells. Notably, skipping of exon 41 does not alter CEP290 protein localisation in control lines C1 and C2. Green - CEP290, Red - ARL13B, Violet - pericentrin. Scale bar 5 μm. (**b**) Immunofluorescence shows that treatment with 5 μM Ex41 skip ASO in serum-free medium for 48 h induces the expression of near full-length CEP290 protein that localises at the same position along the ciliary proximal-distal axis as the TZ component TMEM67. Green - CEP290, Violet - TMEM67, Red - ARL13B. Scale bar 5 μm. (**c**) Immunofluorescence shows that treatment with 5 μM Ex41 skip ASO in serum-free medium for 48 h induces the expression of near full-length CEP290 protein that localises at the same position along the ciliary proximal-distal axis as the TZ component AHI1. Green - CEP290, Violet - AHI1, Red - ARL13B. Scale bar 5 μm. (**d**) Representative immunofluorescence micrographs of control fibroblasts C1, C2 and JBTS5 cells P1 and P2. PCM-1 localisation at centriolar satellites is not altered in the absence of full-length CEP290. Green - CEP290, Violet - PCM-1, Red - ARL13B. Scale bar 5 μm.

In order to evaluate the functionality of the abundant protein produced by exon 41-skipped *CEP290* transcript, we first checked by immunofluorescence whether it correctly localised at the base of the cilium. Serum-starved patient cells treated with Ex41 skip ASO displayed a detectable fluorescent intracellular signal for CEP290 that localises at the base of the cilium (Fig. 5a, Supplementary Fig. S4). To examine the exact localisation of CEP290 we compared it with the position along the ciliary axis of the MKS complex components TMEM67 and AHI1. While in untreated patient cells CEP290 signal at the centriole is absent or faint, the latter case likely due to a read-through of the mutation, in control and treated cells, CEP290 localises at the same position along the ciliary proximal-distal axis as TZ markers and JBTS proteins TMEM61 (JBTS6) and AHI1 (JBTS3, Fig. 5b,c). It is noteworthy that, consistently with that reported previously in JBTS5 cells^17^, there is no obvious defect in the localisation of other TZ proteins in the absence of full-length CEP290 protein, indicating that the overall TZ structure is unaltered (Fig. 5b,c). Similarly, localisation of PCM-1 granules in the pericentriolar region appears unaltered in JBTS5 cells, as well as in cells treated with Ex41 skip ASO, consistent with previous reports^17^ (Fig. 5d).

### Near full-length CEP290 protein expression rescues ciliary defects in JBTS5 fibroblasts

Next, we examined whether Ex41 skip ASO induces the expression of a near full-length CEP290 protein, able to rescue JBTS ciliary defects, including reduced localisation of AC3 and ARL13B proteins in the ciliary membrane, elongated cilia and impaired ciliogenesis.

**Figure 6:**
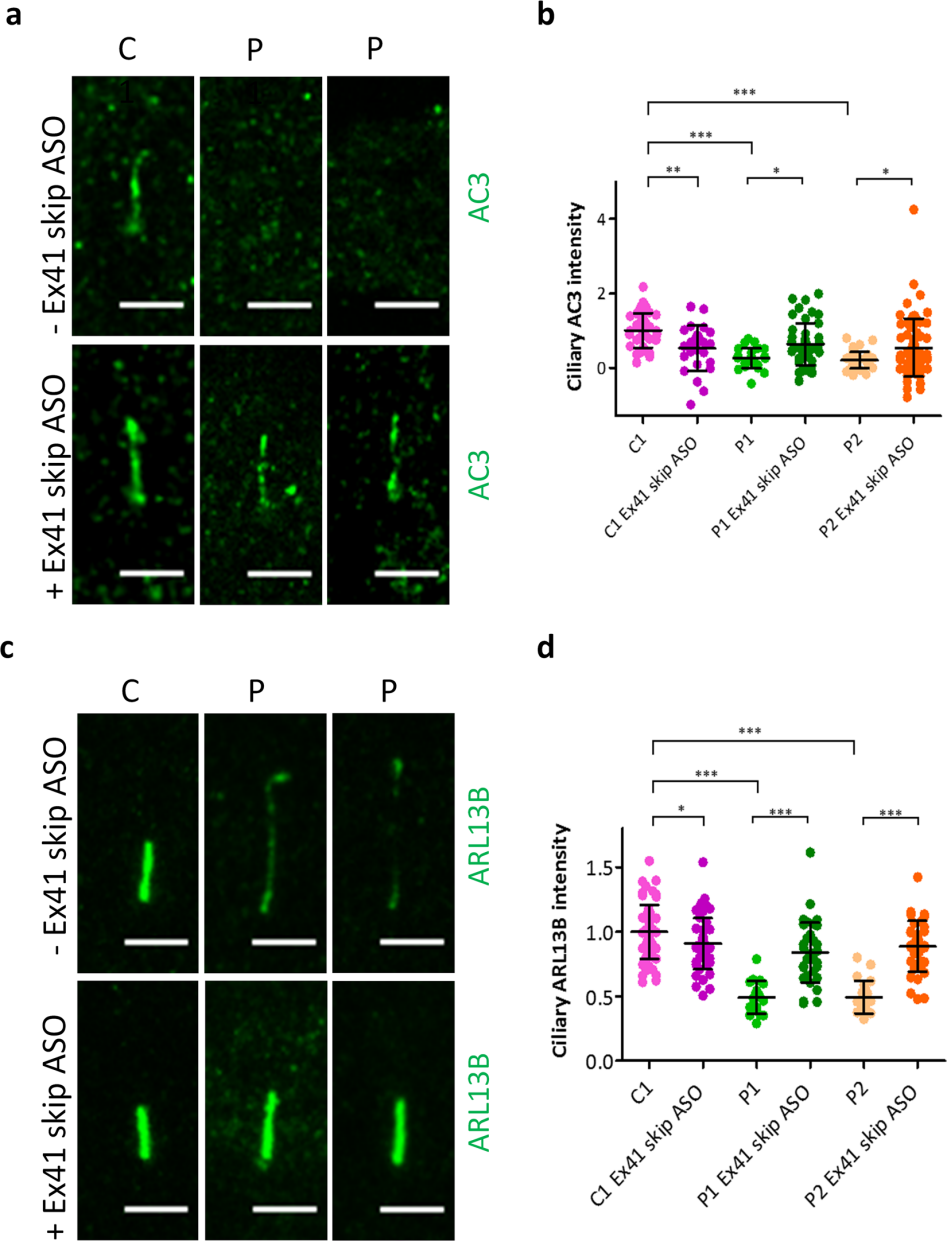
Treatment with Ex41 skip ASO increases ciliary localisation of membrane proteins AC3 and ARL13B in JBTS5 fibroblasts. (**a**) Representative immunofluorescence micrographs of control fibroblasts C1 and JBTS5 cells P1 and P2, serum starved for 48 h. Treatment of JBTS5 fibroblasts with 5 μM Ex41 skip ASO for 48 h increases AC3 ciliary localisation in JBTS5 lines P1 and P2. Green – AC3. Scale bar 5 μm. (**b**) Quantification of ciliary AC3 intensity after background subtraction. Values are expressed as AC3 ciliary intensity relative to ciliary intensity in C1 cells. *P < 0.05, **P < 0.01, ***P < 0.001, one-way ANOVA, n = 248. (**c**) Representative immunofluorescence images of control fibroblasts C1 and JBTS5 cells P1 and P2, serum starved for 48 h. Treatment of JBTS5 fibroblasts with 5 μM Ex41 skip ASO for 48 h increases ciliary localisation of the membrane protein ARL13B. Green – ARL13B. Scale bar 5 μm. (**d**) Quantification of ciliary ARL13B intensity normalised to the background. Values are expressed as ARL13B ciliary intensity relative to ciliary intensity in C1 cells. *P < 0.05, ***P < 0.001, one-way ANOVA, n = 281.

Quantification of AC3 fluorescence signal intensity revealed that AC3 in the cilium significantly increased in Ex41 skip ASO-treated patient fibroblasts (Fig. 6a,b, Supplementary Fig. S5). Similarly, Ex41 skip ASO treatment increased ciliary localisation of ARL13B, implying that the protein product of *CEP290* exon 41-skipped transcript is able to promote correct localisation of membrane-associated proteins to the cilium (Fig. 6c,d, Supplementary Fig. S5).

Hedgehog (HH) signalling defects were shown to be implicated in JBTS^20,21^. qPCR analysis of HH downstream effector *GLI1* demonstrated a strong upregulation (∼14 fold) of HH pathway activation in JBTS5 cells, which was not fully rescued in Ex41 skip ASO treated cells (Supplementary Fig. S6).

**Figure 7:**
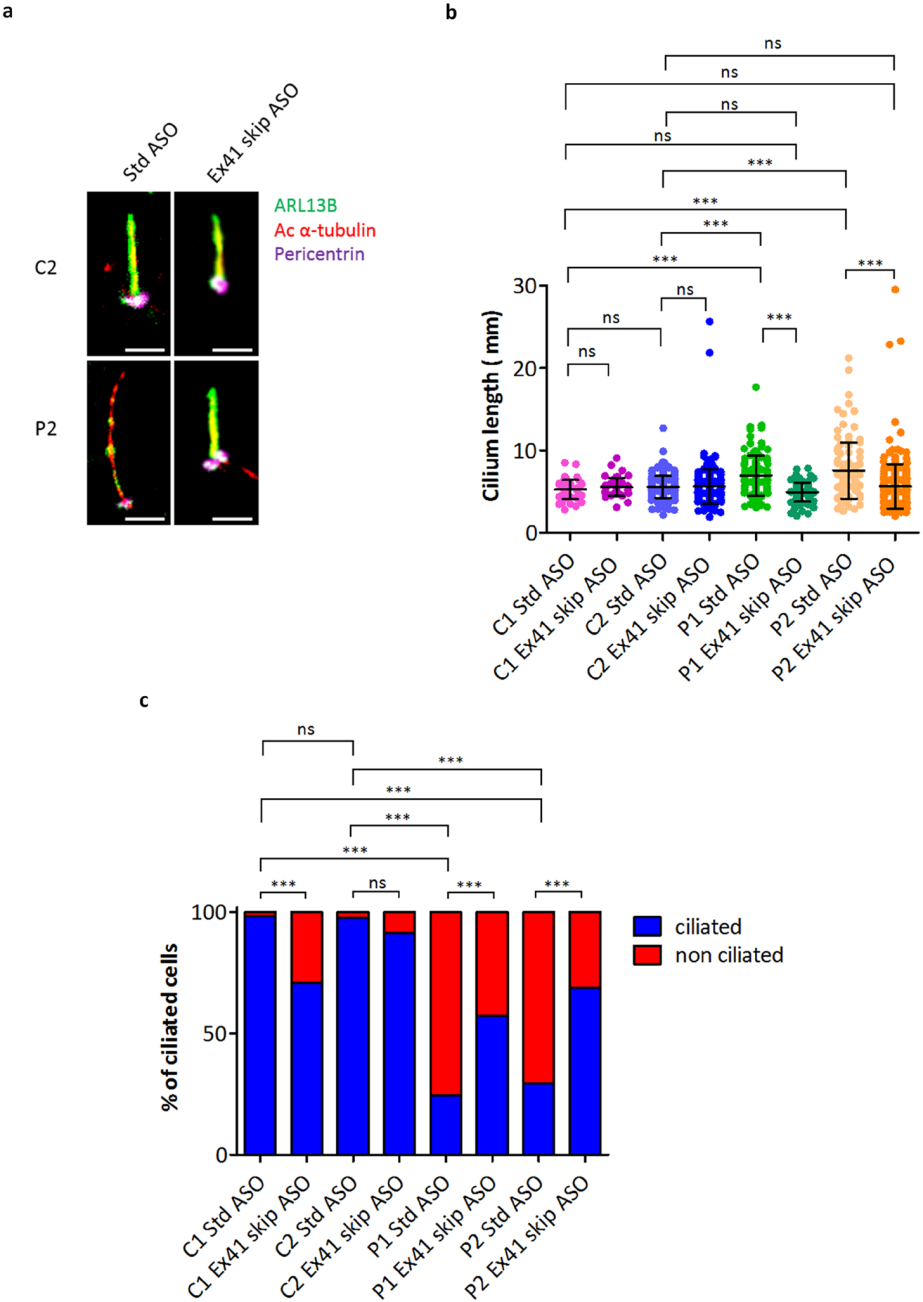
Treatment with Ex41 skip ASO rescues cilia length and biogenesis in JBTS5 fibroblasts. (**a**) Representative immunofluorescence images of control fibroblasts C2 and JBTS5 cells P2, treated with 5 μM Std ASO or 5 μM Ex41 skip ASO and serum starved for 48 h. Treatment with Ex41 skip ASO results in a rescue of ciliary phenotype in JBTS5 cells. Green - ARL13B, Red – acetylated α-tubulin, Violet - pericentrin. Scale bar 3 μm. (**b**) Quantification of cilia length in control fibroblast lines C1, C2 and JBTS5 lines P1, P2, treated with 5 μM Std ASO or 5 μM Ex41 skip ASO. The mean cilia length measured for C1 was 5.3 μm when treated with Std ASO and 5.6 μm after treatment with Ex41 skip ASO. The mean cilia length measured for C2 was 5.6 μm when treated with Std ASO or Ex41 skip ASO. Mean length of JBTS5 fibroblasts P1 cilia decreased from 7.0 μm to a near-control length of 4.9 μm after treatment with Ex41 skip ASO. Similarly, JBTS5 fibroblasts P2 mean cilia length decreased from 7.6 μm to near-control length of 5.7 μm following treatment with Ex41 skip ASO. ***P < 0.001, ns, non-significant, one-way ANOVA, n = 1168, data from three independent experiments. (**c**) Quantification of percentage of ciliated cells in control fibroblast lines C1, C2 and JBTS5 lines P1, P2, treated with 5 μM Std ASO or 5 μM Ex41 skip ASO. The percentage of ciliated cells for C1 was 98% when treated with Std ASO and 71% after treatment with Ex41 skip ASO. The percentage of ciliated cells measured for C2 was 98% when treated with Std ASO and 91% after treatment with Ex41 skip ASO. Percentage of ciliated JBTS5 fibroblasts P1 increased from 24% to 57% after treatment with Ex41 skip ASO. Similarly, the percentage of ciliated JBTS5 fibroblasts P2 increased from 30% to 69%, following treatment with Ex41 skip ASO. ***P < 0.001, ns, non-significant, Fisher’s exact test. Bonferroni correction was used to adjust for multiple comparisons, n = 910.

Immunofluorescence analysis revealed that treatment of JBTS5 fibroblasts P1 and P2 with Ex41 skip ASO reduced cilia length to levels comparable to control cells C1 and C2 (Fig. 7a,b). Finally, ASO treatment also partially rescued cilia biogenesis defects, leading to an increase in the percentage of JBTS5 cells that display a primary cilium after serum starvation (Fig. 7c, Supplementary Fig. S6).

## Discussion

### An array of ciliary defects characterises JBTS5 fibroblasts

Primary cilia are important signalling hubs in which coordination and extensive cross-talk between multiple signals from the extracellular environment take place. Dysfunctional primary cilia may lead to impaired signal transduction with dramatic consequences on tissue development and homeostasis^22^. In order to identify essential phenotypic end-points to predict efficacy of potential therapies *in vivo*, it is important to understand how JBTS mutations affect ciliary morphology, composition and signalling.

CEP290 is required for assembly of the primary cilium and siRNA knockdown in human cells results in a reduction of ciliogenesis^13,14^. Consistent with this, we show that JBTS5 fibroblasts, characterised by low *CEP290* expression levels, display cilium biogenesis defects. Lack of *CEP290* expression in JBTS5 fibroblasts also results in an elongated ciliary phenotype. This phenotype is consistent with that previously reported in JBTS5 URECs^18,23^ and fibroblasts^17^. On the other hand, URECs and fibroblasts derived from patients with Leber congenital amaurosis (LCA), a severe form of retinal degeneration with no renal or cerebellar manifestations, displayed detectable levels of full-length CEP290, normal ciliogenesis and cilia morphology^17,23^. Taken together, these results indicate that aberrant cilium biogenesis and length may be characteristic features of JBTS cells and suggest a possible link between expression level of CEP290, ciliary phenotype and severity of clinical presentation.

It has been demonstrated that TZ proteins are required to preserve TZ structure integrity and trafficking of ciliary membrane proteins, which are involved in ciliary signalling^4,5,8^. MEFs null for JBTS genes that belong to the TZ MKS complex display a reduction of ciliary enrichment of the membrane proteins AC3 and ARL13B^4^. Consistent with the gating function of TZ proteins to control ciliary membrane composition, fibroblasts from a single JBTS5 patient were shown to be defective in AC3 ciliary localisation^17^. To understand whether the defective ciliary localisation of AC3 is a common feature of JBTS5 fibroblasts, we investigated the localisation of AC3 to the cilium of fibroblasts in two different JBTS5 patients. Indeed, our results show that low expression levels of CEP290 in JBTS5 fibroblasts lead consistently to defects in ciliary membrane composition. Similarly, we demonstrate that low levels of CEP290 in P1 fibroblasts result in a reduction of ARL13B ciliary localisation, as previously reported for P2^17^, confirming that mislocalisation of ciliary proteins is a widespread feature in JBTS cells.

We observed a strong upregulation of HH signalling upon SAG treatment in JBTS5 cells compared to control cells, even though the number of cilia was lower. This observation is in line with that previously observed in JBTS5 cells^17^ and points toward the fact that CEP290 is a negative regulator of HH signalling. We hypothesise that decreased levels of AC3 within the cilium, due to disturbed membrane protein trafficking, may contribute to the observed upregulation of HH in JBTS5 fibroblasts. In fact, it has been shown that ACs regulate HH pathway in mouse cerebellar granular neuron precursors and in chicken embryonic neural tube, where their ciliary localisation is required to repress HH signalling^24^, likely through the regulation of Protein kinase A (PKA) activity at the cilium base. Altered ciliary trafficking following pathway activation of other HH regulators, such as GPR161 and SMO, in the absence of a functional CEP290, is likely as well to contribute to the observed upregulation of *GLI1* levels, as shown in fibroblasts from a different JBTS5 individual^17^.

Despite the observed alterations in ciliary membrane protein trafficking, which is consistent with a loss of TZ diffusion barrier function, the lack of full-length CEP290 protein doesn’t result in a disruption of TZ structure, as indicated by the normal localisation of MKS complex proteins TMEM67 and AHI1 in JBTS5 cells. The preservation of TZ structure integrity in the absence of CEP290 in patient cells is consistent with previous studies in human *CEP290* mutant cells^17^ but appears to be in contrast with observations in a *cep290 Caenorhabditis elegans* mutant strain, where lack of cep290 results in an impaired assembly of the MKS module at the TZ^25^. These results may underlie a species-specific role of CEP290 that, in human cells, appears to be necessary for maintaining TZ diffusion barrier function but, unlike other TZ components such as TCTN2 and RPGRIP1L^5^, is not essential for TZ structural integrity.

### Treatment with forskolin partially rescues the ciliary defects in JBTS5 fibroblasts

It has been shown that AC3 inhibition can result in aberrant ciliary elongation and that pre-treatment with forskolin can partly prevent elongation induced by lithium-mediated inhibition of AC3^19^. Consistently, we show here that treatment of JBTS5 fibroblasts with AC activator forskolin results in shortening of cilia to levels comparable to healthy controls. We therefore propose that activation of residual ciliary AC3 with forskolin may partially restore its function in JBTS5 fibroblasts, rescuing aberrant cilia elongation.

Indeed, modulation of cyclic AMP (cAMP) levels, the downstream product of AC3, in the treatment of renal ciliopathies has been widely studied with remarkable success and the V2 receptor antagonist tolvaptan is the only approved non-conservative treatment for the ciliopathy autosomal dominant polycystic kidney disease^26^. However, JBTS5 fibroblasts display an array of ciliary defects, some of which do not seem to be downstream of AC3 mislocalisation and thus cannot be rescued by its activation. ARL13B is reduced in the ciliary membrane of JBTS5 fibroblasts and this defect cannot be rescued by forskolin treatment, indicating that it may be a direct consequence of loss of CEP290 gate-keeper function at the TZ.

### Targeted exon skipping restores CEP290 functions at the TZ in JBTS5 fibroblasts

As opposed to pharmacological interventions that target a particular aberrant pathway associated with the disease, correction of the primary genetic lesion represents a more efficient means to restore the full array of *CEP290* functions, without the risk of unwanted side effects.

However, due to the large gene size, gene replacement therapies may prove challenging in the case of *CEP290*, although lentiviral transfer of *CEP290* has been employed successfully in patient cells^27^ and administration of multiple AAV intein vectors improves the retinal phenotype of LCA mice by reconstituting full-length CEP290 expression^28^. Another strategy that has been successfully explored in LCA models to overcome the limitations imposed by *CEP290* large size is represented by the *in trans* complementation of the mutation using selected fragments of the gene^29,30^. On the other hand, mutations that affect transcript splicing would be ideal targets for ASO-mediated therapies, as shown in the case of the common mutation c.2991 + 1655A > G in *CEP290*, which introduces a pseudo-exon and causes LCA^31–33^. Importantly, a recent clinical trial has demonstrated safety and tolerability of intravitreal injections of ASO, to correct aberrant splicing and showed improved visual acuity in these patients^34^.

Interestingly, several studies have recently shown in retinal dystrophy patients, with nonsense mutations in *CEP290*, that mild retinal phenotypes are associated with nonsense-mediated alternative splicing or endogenous basal exon skipping^35,36^. It has been proposed that a widespread, low-level alternative splicing leads to the expression of low levels of transcripts lacking the mutated exon, which can mitigate the effects of certain mutations of *CEP290*, partially explaining the observed genetic pleiotropy^37^.

Indeed, qRT-PCR on fibroblast cDNA from a patient carrying a truncating mutation on exon 41 revealed high levels of *CEP290* transcript lacking this exon, when normalised to total *CEP290* transcript, compared to control or other patients with different mutations in *CEP290*^37^. This indicates that skipping of exon 41 occurs naturally to a certain extent and prevents degradation of skipped transcript.

We have recently shown that targeted skipping of the mutated exon 41 is a promising therapeutic approach for JBTS5, as it restores CEP290 near full-length protein expression and rescues elongated cilia in URECs from a JBTS5 patient homozygous for the mutation c.5668 G > T; p.(G1890X) in *CEP290*. Moreover, we showed that systemic delivery of an ASO to skip the gene-trap in a mouse model of JBTS5 leads to a rescue of elongated cilia and cystic phenotype in the kidney^18^.

Here we expand our studies to investigate at a molecular level the consequences of ASO-mediated restoration of CEP290 expression and show a rescue of TZ functions, whose loss has been proposed to be at the basis of JBTS^5^. We show that ASO treatment results in a restoration of gate-keeper functions of CEP290 protein in JBTS5 patient fibroblasts, with a rescue not only of cilia biogenesis and morphology, but also of ciliary localisation of AC3 and ARL13B. Moreover, by testing ASO-mediated exon skipping in fibroblasts from a compound heterozygote patient that carries a skippable mutation on a single allele, we show that ASO is consistently able to rescue ciliary defects in multiple patients and that the skipping of heterozygous allele of a compound heterozygous phenotype is sufficient to induce the rescue.

To date, there is no definitive treatment for JBTS, despite the severity of associated symptoms, such as retinal degeneration and nephronophthisis, which can result in end-stage renal failure at a median age of 13 years. The slowly progressive renal disease leaves time in many cases for therapeutic interventions, such as ASO treatment targeted towards the kidney.

ASO-based therapeutic approaches to promote targeted exon skipping and restore transcript reading frame have been successfully tested in other genetic conditions. Eteplirsen, an ASO with a morpholino backbone, has recently received accelerated approval from the FDA for the treatment of Duchenne muscular dystrophy^38^.

Further *in vivo* studies on humanised mouse models will be required to better assess the consequences of exon skipping on renal and extra-renal tissues and precision medicine approaches are warranted to evaluate the suitability of exon skipping to treat patients with different mutations.

However, the functional *in vitro* study that we present here demonstrates the enormous potential of this strategy to restore TZ functions altered in JBTS5 human cells, providing an encouraging picture for the translational value of this therapeutic approach.

## Methods

### Fibroblast culture

Following informed and written consent, skin samples were obtained from JBTS5 patients and healthy controls. Ethical approval was given by the National Research Ethics Service Committee North East – Newcastle and North Tyneside 1 (08/H0906/28+5) and the National Research Ethics Service (NRES) Committee North East (14/NE/1076). All methods were performed in accordance with the relevant ethical guidelines and regulations.

Skin biopsies were stored in Ham’s F-10 complete medium (100 ml Ham’s F-10), 20 ml FCS (Seralab), 2 ml Penicillin/Streptomycin solution (10,000 U/ml), 1 ml GlutaMAX 100 × (Thermo Fisher), 1 ml Fungizone (Thermo Fisher) at 4 °C for 24 h. The following day, biopsies were washed in PBS and a 3 mm^2^ fragment of skin was cut using scalpels. After centrifugation in 2.5% Trypsin, tissues were incubated for 15 min at 37 °C and further centrifuged for 5 min. Then trypsin was carefully removed and tissues were incubated in collagenase type IV solution (100 mg in 100 ml Ham’s F-10) for 90 min at 37 °C. After centrifugation, collagenase was removed and replaced with Ham’s F-10 complete medium and cell suspension was placed into a T25 flask and incubated at 37 °C in 5% (v/v) CO2 in a humidified incubator.

After 48 h, medium was carefully removed and replaced with fresh medium. The medium was changed every three days. Once fibroblasts attained 70% confluence, the cells were washed in PBS, dissociated with TrypLE Express (Thermo Fisher) and transferred into 2 T75 flasks and returned to incubator.

Fibroblasts were kept in DMEM supplemented with 10% FBS (Thermo Fisher) and Penicillin/Streptomycin solution (10,000 U/ml, Thermo Fisher) 1:100 at 37 °C in a humidified atmosphere of 5% (v/v) CO_2_ and passaged when reaching 80% confluence.

70,000 cells were seeded on top of a 13 mm diameter glass coverslip for immunofluorescence applications, alternatively 600,000 cells were seeded in a T25 flask for RNA extraction, or 200,000 cells were seeded in each well of a 12 well plate for protein extraction.

### Antisense oligonucleotide (ASO) treatments

To induce skipping of exon 41 from *CEP290* transcript, a morpholino ASO (Ex41 skip ASO) (GeneTools LLC, USA) was designed to target the intron-exon boundary of human *CEP290* (exon 41-intron 41, 5′-ATGTTTCTTCACATACCTTTTCTTT-3′). Ex41 skip ASO was dissolved in RNase-free water to make a 1 mM stock solution. A Standard control ASO (Std ASO) with no target sequence in the human genome (5′-CCTCTTACCTCAGTTACAATTTATA-3′) was used as a control. 24 h after seeding, fibroblasts were treated with 6 μM Endo-Porter (GeneTools LLC, USA) and with the indicated concentrations of Std ASO or Ex41 skip ASO in serum-free medium for 48 h.

### Drug treatments

Where indicated, fibroblasts were treated with 25 μM forskolin (Selleckchem)/0.1% DMSO (Sigma-Aldrich) in serum-free medium for 48 h before analysis or with 100 nM SAG (Tocris)/0.1% DMSO (Sigma-Aldrich) in serum-free medium for 24 h before analysis.

### Immunofluorescence imaging

Fibroblasts were fixed in ice-cold methanol for 10 min. After 30 min saturation with 5% BSA in PBS, cells were incubated for 1 h at room temperature with the following primary antibodies in blocking solution: rabbit anti-ARL13B (Proteintech, 17711-1-AP); mouse anti-ARL13B (Proteintech, 66739-1-Ig); rabbit anti-CEP290 (Abcam, ab 85728); mouse anti-Pericentrin (Abcam, ab28144); rabbit anti-Pericentrin (Abcam, ab 4448); mouse anti-acetylated α-tubulin (Sigma, T6793); rabbit anti-AC3 (Abcam, ab 123803); rabbit anti-TMEM67 (Proteintech, 13975-1-AP); rabbit anti-AHI1 (Proteintech, 22045-1-AP); rabbit anti PCM-1 (Santa Cruz, sc-67204). Following washes in PBS, cells were incubated at room temperature for 1 h with the following secondary antibodies: donkey anti-rabbit Alexa Fluor 488 (Thermo Fisher); donkey anti-mouse Alexa Fluor 594 (Thermo Fisher); goat anti-mouse Alexa Fluor 647 (Thermo Fisher). Alternatively, cells were incubated overnight at 4 °C with primary rabbit antibodies directly labelled using Zenon Alexa Fluor rabbit IgG labelling kit (Thermo Fisher), washed with 0.1% PBS Tween and post-fixed with 4% PFA for 15 min. Following final washes in PBS, cells were mounted in Vectashield (Vector Laboratories Ltd, H-1200). Images and *z*-stacks were captured in a blinded fashion, using a Nikon (A1) confocal inverted microscope. Laser power was kept constant within each experiment for the channels used in intensity measurements. If channels were not used for intensity measurements, laser power was adjusted occasionally to facilitate cilia localisation.

### Scanning electron microscopy (SEM) imaging

For SEM, samples were fixed overnight in 2% glutaraldehyde in 0.1 M Sorenson’s phosphate buffer, dehydrated through a graded series of ethanol and then critical-point dried (Baltec dryer).

They were coated with 10 nm of gold (Polaron coating unit) and viewed on a Tescan Vega LMU SEM operated at 8–10 kV.

### Image analysis

Following capture, images were analysed using FIJI (ImageJ) software. The length of cilia was measured using the segmented line tool on a maximum intensity projection of a *z*-stack, merge of ARL13B and acetylated α-tubulin channels was used to identify the cilia and ARL13B was used to quantify cilia length. Fluorescence intensity of individual cilia was measured on a sum of slices projection of a *z*-stack; a region of interest (ROI) was constructed around the cilia (identified with ARL13B staining or acetylated α-tubulin staining) to measure the mean grey value. To correct for local background intensity, the ROI was duplicated and dragged to a nearby region and background fluorescence intensity was measured.

### Western blotting

A lysis buffer solution containing 4 M urea, 125 mM Tris pH 6.8, 4% SDS, 10% glycerol, 5% β-mercaptoethanol and 0.02% bromophenol blue was used to lyse fibroblast cells. Protein samples were then heated to 95 °C for 5 min, spun at 16 000 rcf for 5 min and resolved by SDS-PAGE on a 4–20% gradient gel (Bio-Rad). Proteins were transferred to a 0.4 µm nitrocellulose membrane (Thermo Fisher). The membranes were blocked in TBS Tween (Tris-buffered saline, 0.1% Tween-20) containing 5% low fat milk for 1 h, then incubated with the following primary antibodies in blocking solution overnight at 4 °C: rabbit anti-CEP290 (Abcam, ab 85728); rabbit anti ARL13B (Proteintech, 17711-1-AP); rabbit anti-AC3 (Abcam, ab 123803); mouse anti-β-Actin (Sigma, A228), rabbit anti-GAPDH (CST, 14C10), mouse anti-Vinculin (Sigma, V9131). After washing in TBST, membranes were incubated with fluorescently labelled secondary antibodies (LI-COR) for 90 min at room temperature, washed again in TBST and visualized with an Odyssey CLx imaging system (LI-COR).

### RT-PCR and qRT-PCR

RNA was extracted from fibroblast cells after 48 h serum starvation using RNeasy minikit (Qiagen) according to the manufacturer’s instructions and quantified using a NanoDrop 2000 Spectrophotometer (Thermo Fisher). 1 μg of RNA was reverse-transcribed using an Oligo-dT primer and SuperScript III Reverse Transcriptase (Thermo Fisher). The resulting cDNA was diluted 10-fold in nuclease-free water. RT-PCR, using GoTaq® DNA Polymerase (Promega) and *CEP290* gene-specific primer pairs (5- TTTTAGAACTCCGGGCAGAA-3 and 5-TTGGCTTGCCACTTTTTACC-3), was performed to identify splice products following ASO treatment. Amplification of *HPRT1* housekeeping gene cDNA was performed alongside.

qPCR to determine *CEP290* expression was carried out using PrimeTime^®^ Gene Expression Master Mix (IDT) and the PrimeTime^®^ qPCR probe-based assay (IDT) CEP290-Hs.PT.58.2324908 (IDT) according to manufacturer’s instructions on cDNA diluted 5-fold. Expression levels were normalised to the expression of housekeeping genes *HPRT1* and *GAPDH*.

Quantitative analysis of *GLI1* expression was carried out using TaqMan probe Hs00171790_m1 (Thermo Fisher) and the qScript XLT one-step RT-qPCR mix (Quantabio) according to manufacturer’s instructions on RNA diluted 5-fold. Expression levels were normalised to the expression of housekeeping genes *HPRT1* and *GUSB*.

### Statistics

All data are shown as the mean ± standard deviation. One-way ANOVA followed by a Newman-Keuls Multiple Comparison Test to correct for multiple comparisons was performed, unless otherwise stated. A *P* value of less than 0.05 was considered statistically significant.

## Supplementary information

Supplementary

## Data Availability

The data generated and further information on the materials used in the current study are available from the corresponding author on request.
