# Supplementary material for: Targeted exon skipping rescues ciliary protein composition defects in Joubert syndrome patient fibroblasts
Source: Sci Rep. 2019 Jul 25;9:10828. doi: 10.1038/s41598-019-47243-z (PMC6658666; doi:10.1038/s41598-019-47243-z)
Supplement: Supplementary file 1 — Supplementary [file 41598_2019_47243_MOESM1_ESM.pdf]

Supplementary Information

Targeted exon skipping rescues ciliary protein composition defects in Joubert syndrome patient fibroblasts

Elisa Molinari, Simon A. Ramsbottom, Shalabh Srivastava, Philip Booth, Sumaya Alkanderi, Seamus M. McLafferty, Laura A. Devlin, Kathryn White, Meral Gunay-Aygun, Colin G. Miles & John A. Sayer

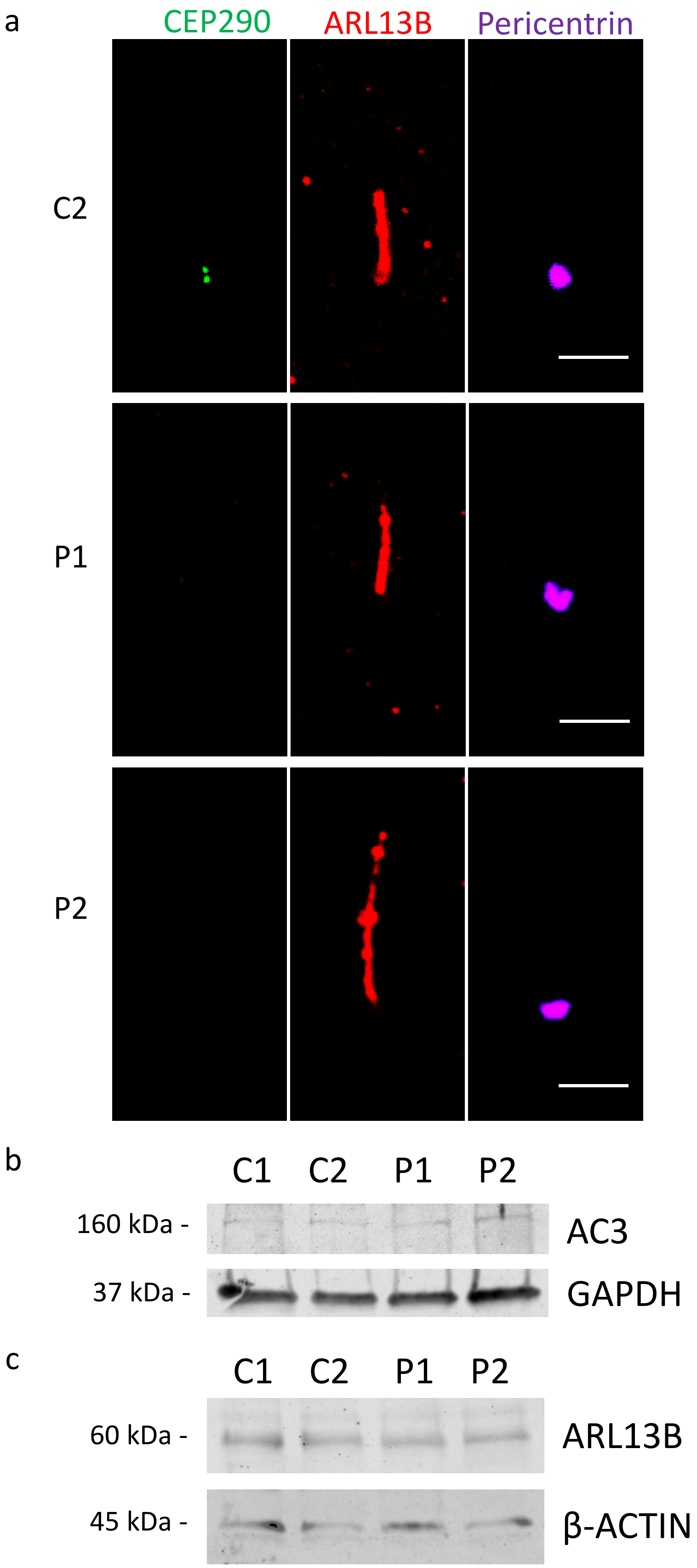

Supplementary Fig. S1. (a) Single channel view of immunofluorescence microscopy of control heterozygous fibroblasts C2 and JBTS5 fibroblasts P1 and P2, serum starved for 48 h. CEP290 protein is visible at the base of the cilium in control fibroblasts, but is not detectable in P1 and P2. Green - CEP290, Red - ARL13B, Violet - pericentrin. Scale bar 5μm. (b) Western blot shows that AC3 total protein levels are not reduced in P1 and P2 cells compared to control cells. GAPDH serves as loading control. (c) Western blot shows that ARL13B total protein levels are not reduced in JBTS5 fibroblasts compared to control cells. B-ACTIN serves as loading control.

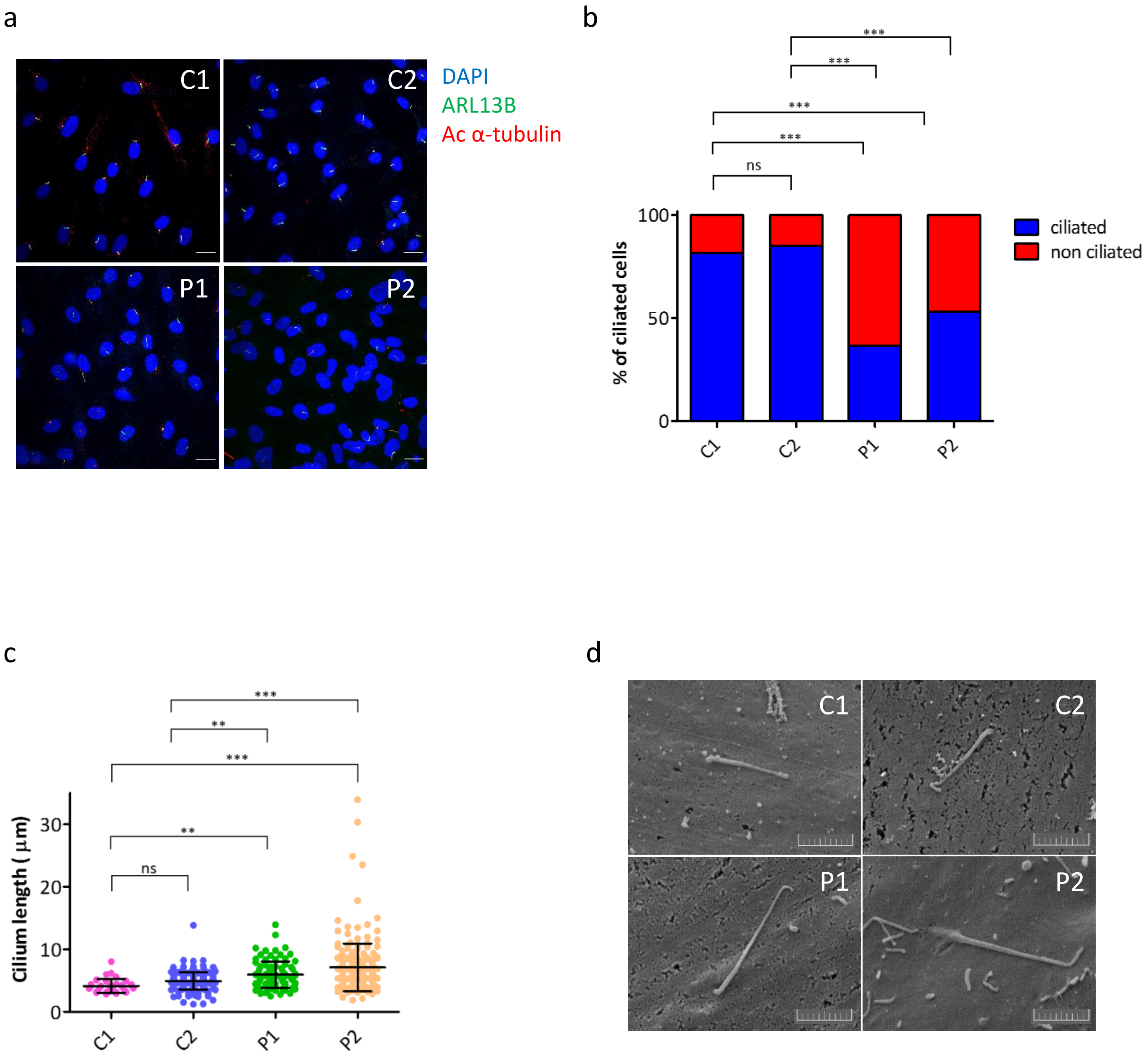

Supplementary Fig. S2. (a) Immunofluorescence microscopy of control fibroblast lines C1, C2 and JBTS5 lines P1, P2, serum starved for 48 h, reveals impaired ciliogenesis and highly variable cilia length in JBTS5 cells. DAPI- blue (Nuclei), Green - ARL13B, Red - Acetylated α-tubulin. Scale bar 20µm. (b) Quantification of percentage of ciliated cells in control fibroblast lines C1, C2 and JBTS5 lines P1, P2. ***P < 0.001, ns, non-significant, Fisher's exact test. Bonferroni correction was used to adjust for multiple comparisons, n = 735. (c) Quantification of cilia length in immunofluorescent micrographs of control fibroblast lines C1, C2 and JBTS5 lines P1, P2, after 48 h starvation. JBTS5 cilia are highly variable in length and are on average longer than control cilia. **p < 0.01, ***P < 0.001, ns, non-significant, one-way ANOVA, n = 573, data from three independent experiments. (d) Scanning electron microscopy of control fibroblast lines C1, C2 and JBTS5 lines P1, P2, serum starved for 48 h, confirms elongated phenotype in JBTS5 cilia. No altered thickness of the cilia is observable, indicating that reduction in ARL13B fluorescent signal is not due to thinning of ciliary structure, but to a reduction of ciliary ARL13B levels. Scale bar 2 µm.

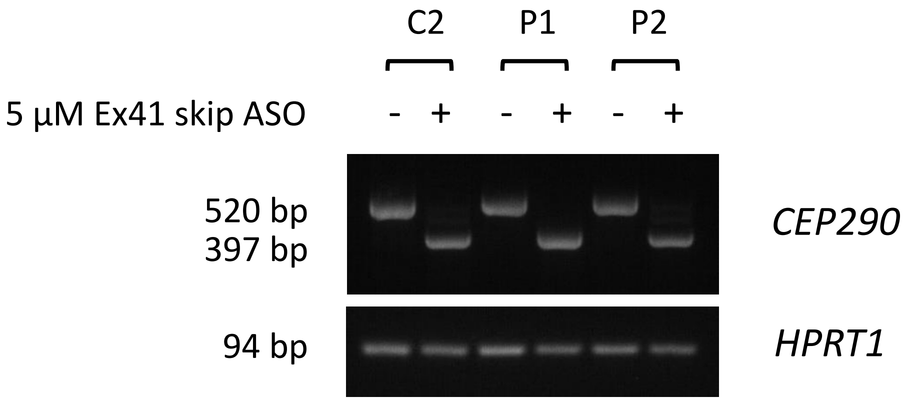

Supplementary Fig. S3. RT-PCR on cDNA isolated from C2, P1 and P2 lysates untreated or treated with 5 µM Ex41 skip ASO for 48 h. Treatment with 5 µM Ex41 skip ASO leads to the skipping of 123 bp of exon 41 in the totality of *CEP290* transcript (PCR product length, 397 bp). In untreated cells only full-length *CEP290* transcript is detectable (PCR product length, 520 bp). *HPRT1* was used as housekeeping gene.

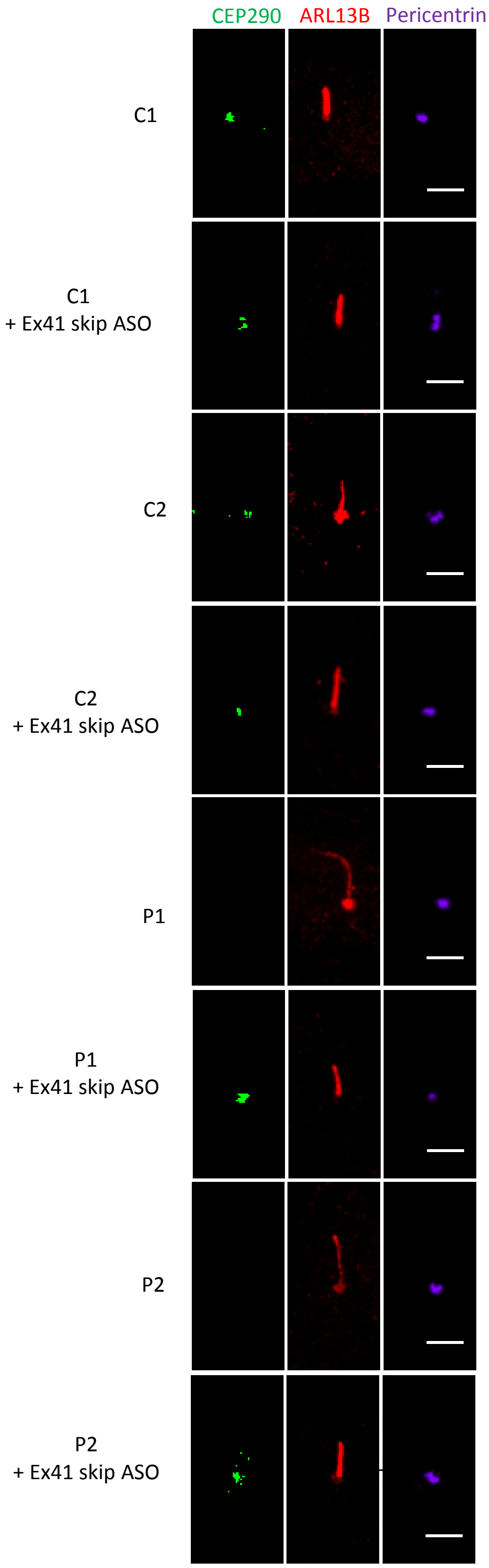

Supplementary Fig. S4. Single channel view of immunofluorescence microscopy showing that CEP290 protein is not detectable in untreated P1 and P2 cells, serum starved for 48 h. Treatment with 5 µM Ex41 skip ASO rescues CEP290 protein expression which localises to the base of the cilium, in P1 and P2 cells. Green - CEP290, Red - ARL13B, Violet - pericentrin. Scale bar 5µm.

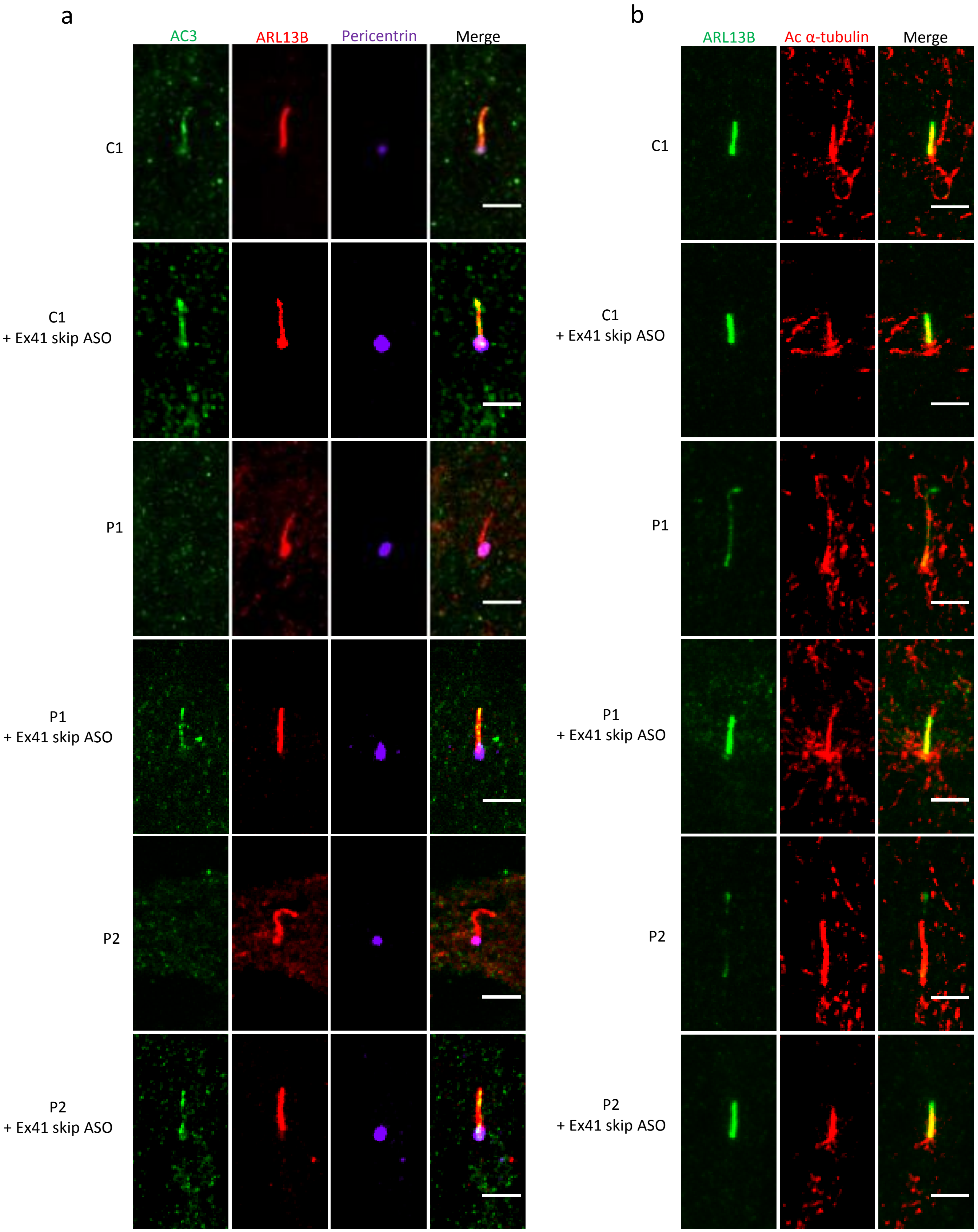

Supplementary Fig. S5. (a) Single channel and merged view of immunofluorescence microscopy of control fibroblasts C1 and JBTS5 cells P1 and P2, serum starved for 48 h. Treatment of JBTS5 fibroblasts with 5 µM Ex41 skip ASO for 48 h increases AC3 ciliary localisation in JBTS5 lines P1 and P2. Green – AC3, Red – ARL13B, Violet – pericentrin. Scale bar 5µm. (b) Single channel and merged view of immunofluorescence microscopy of control fibroblasts C1 and JBTS5 cells P1 and P2, serum starved for 48 h. Treatment of JBTS5 fibroblasts with 5 µM Ex41 skip ASO for 48 h increases ciliary localisation of the membrane protein ARL13B. Green – ARL13B, Red – acetylated α-tubulin. Scale bar 5µm.

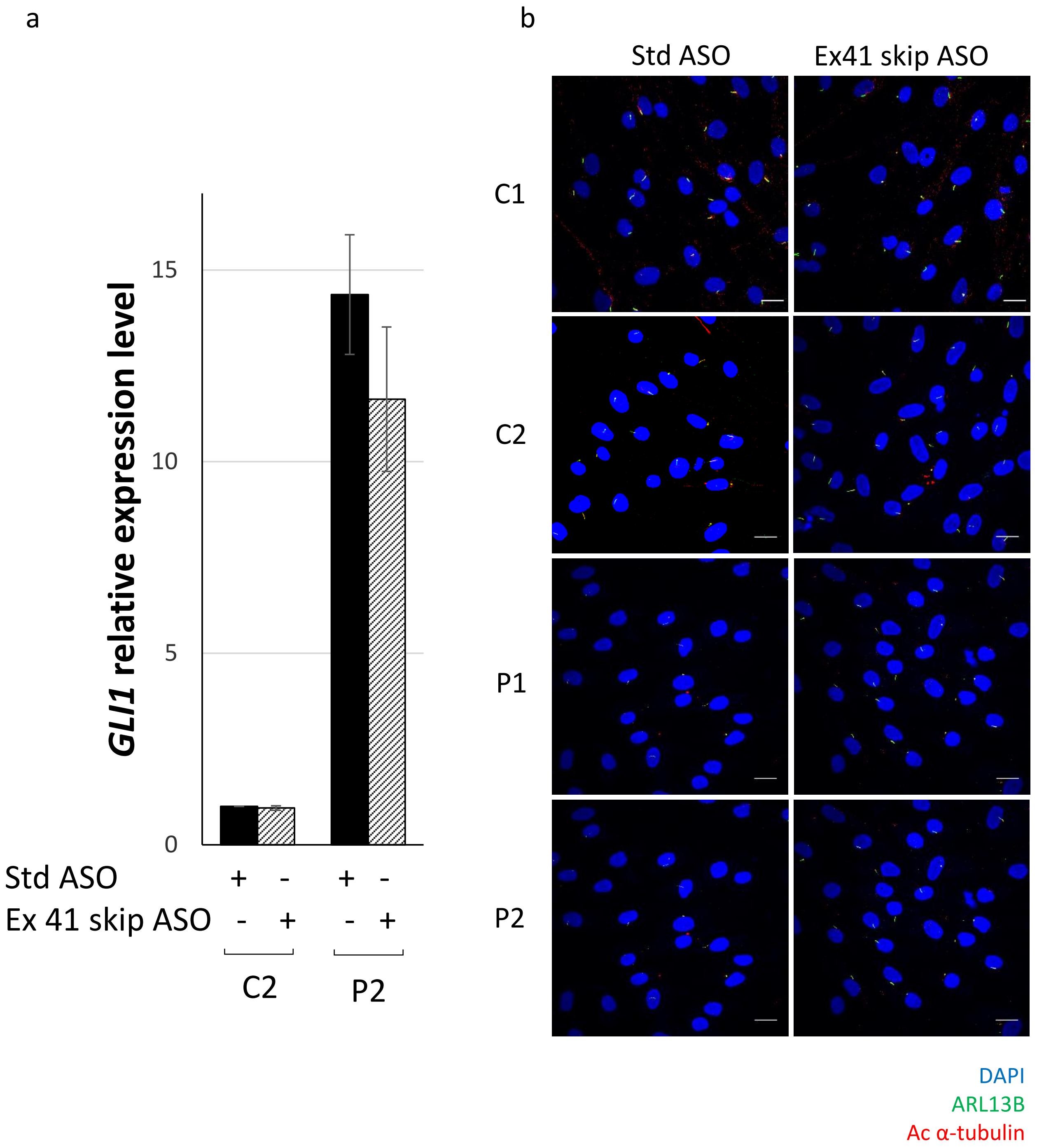

Supplementary Fig. S6. (a) *GLI1* gene expression analysis of fibroblasts obtained from control heterozygote fibroblasts C2 and JBTS5 fibroblasts P2 treated with 5 µM Std ASO or 5 µM Ex41 skip ASO, serum starved for 48 h and treated with 100nM of the HH agonist SAG for 24 h. *GLI1* levels are strongly upregulated (~14 fold) in P2 cells, treatment with Ex41 skip ASO leads to a partial 20% decrease of *GLI1* expression levels in P2 cells. Each bar represents mean value from three replicates. Values are normalised to heterozygote control C2. (b) Immunofluorescence microscopy of control fibroblast lines C1, C2 and JBTS5 lines P1, P2, treated with 5 µM Std ASO or 5 µM Ex41 skip ASO in serum-free medium for 48 h. Treatment with Ex41 skip ASO results in an increase of ciliogenesis in JBTS5 cells. DAPI- blue (Nuclei), Green - ARL13B, Red - Acetylated α-tubulin. Scale bar 20µm.

Supplementary Table S1. Genetics of patients and controls

	Genotype	Alterations in protein sequence	Mutated exons	Families	Sex
**Control C1**	Wild type	-	-		F
**Control C2**	Het c.5668 G>T	Het p.(G1890X)	Exon 41	Mother of P2	F
**Patient P1**	Hom c.5668 G>T	Hom p.(G1890X)	Exon 41		M
**Patient P2**	Het c.5668 G>T,<br>Het c.2495_2512delInATCT	Het p.(G1890X)<br>Het p.(T832Nfs*12)	Exon 41<br>Exon 24	Daughter of C2	F
